# Practical Oral Therapeutics

**Published:** 1894-04

**Authors:** Geo. V. Brown

**Affiliations:** Duluth, Minn.


### Practical Oral Therapeutics.

BY GEO. V. BROWN, M.D., DULUTH, MINN.

Read in the Section on Dental and Oral Surgery, at the Forty-fourth Annual Meeting of the American Medical Association.

Genius has been defined by Carlyle as "an immense capacity for taking trouble," and the methods of oral therapeutics require genius in just that sense for their administration.

Having in charge the natural gateway of the whole wonderfully complex system of human mechanism, we are familiar through experience and the frequent statements in medical and dental literature, with the fact that inflammations having their exciting irritation in the mouth often cause by reflex action severe neuralgic pain in the eye, ear, different parts of the head, face and neck, or whenever connection can be found through the many ramifications of the fifth nerve and its ganglionic associates, that in the same manner also sometimes originate diseased conditions of a very serious nature in the eye and ear, as well as spasmodic affections of the muscles, lockjaw, convulsions, epileptic attacks, paralysis, nervousness and indigestion, and that too there are in contra-distinction to these another class to which belong the infectious diseases of the oral cavity itself, and those that are caused by the migration of pathogenic micro-organisms from the mouth to other parts of the body.

Miller, in his "Micro-organisms of the Human Mouth," considers the diseases caused by the pathogenic bacteria of the mouth under six heads according to the point of entrance of the infection, and it is this classification which I shall follow in discussing their treatment:

1. Infections caused by a breach in the continuity of the mucous membrane by mechanical injuries (wounds).

2. Infections through the medium of gangrenous tooth pulps and from alveolar abscess.

3. Disturbances conditioned by the resorption of poisonous waste products formed by bacteria.

4. Pulmonary diseases caused by the inspiration of particles of slime, small pieces of tartar, etc., containing bacteria.

5. Excessive fermentative processes, and other complaints of the digestive tract, caused by the continued swallowing of microbes and their poisonous products.

6. Infections of the intact soft tissues of the oral and pharyngeal cavities whose power of resistance has been impaired by debilitating diseases, mechanical irritation, etc., considering in this connection also the possibilities of infection by the accumulation in the mouth of the excitants of diphtheria, typho-syphilis and diseases of like nature.

Of the affections not caused by pathogenic bacteria, by far the greater number are the result of an inflammation of the tooth pulp, discussion of the treatment of which in all the variety of opinions so frequently brought forward by the enthusiastic adherents of each, would, of course, be quite beyond the possibilities of this paper. Therefore, I will simply state that in this opinion, while fully recognizing the important value of the pulp in a healthy condition, once having been irritated by exposure to external influences and afterward covered by a capping, it must always afterward be looked upon as a source of danger and a menace to the associated parts, unless the circumstances be so favorable that success is assured.

By the careful destruction and removal of the contents of the pulp chamber and canals, the thorough cleansing with antiseptic measures, and the filling of the roots with gutta-percha, inflammations of this character may be promptly and efficiently relieved. Once the exciting cause has been discovered and removed, usually nothing further is necessary in this class of inflammatory processes unless it might be the use of an astringent, a counter-irritant, or better still where practicable, the lance.

In taking up the treatment of diseases caused by pathogenic bacteria of the oral cavity, a rapid glance at the possibilities in this direction seems to be demanded.

The toxic properties of the human saliva have been noticed by observers since the earliest times, and the experiments of modern biologists have fully borne out the truth of their reports.

Fatal results, attendant upon the bite of persons, and the death of animals injected with human saliva, for the purpose of experimentation, have been explained, as we all know, by the transmission into the circulation of the omnipresent pathogenic bacteria, finding as they do in the mouth, a home easy of access, and possessing all of the little conveniences in suitable temperature, moisture and proper nourishment that go to make the microbe's lot a happy one.

Of primary importance, then, must be the care of our hands and instruments. Fatal septicemia, pyemia, and the transmission of syphilis through accidental wounding of the mouth with infected instruments are too well understood to need more than a passing reference here.

In my own practice it is my custom to keep a small jar of $\frac{1}{1000}$ preparation of bichlorid of mercury upon the operating table, and I have given myself the habit of dipping my mouth mirror and every instrument that I use into it before putting it into the mouth. One has the comfortable feeling that an instrument subject to the corroding influence of bichlorid, and then wiped so thoroughly that it does not corrode is at least mechanically cleaned, even if the action of the germicide might be questionable in so short a period of time.

In using instruments upon the soft tissues and upon the bony structures, I take the additional precaution of an open flame, because even if there be damage to the temper of the instrument it does not seriously unfit it for use in this manner.

The care of the necks of the teeth and that most obstinate affection at the gingival margin known under the various names of *pyorrhea alveolaris*, *Rigg's disease*, *phagedenic pericementitis* etc., on through a variety of different appellations, each, however, signifying a chronic suppurative inflammation of the periosteum accompanied by an inflamed condition of the gums, and more or less affection of the alveolar process.

Undoubtedly there are constitutional predisposing conditions which are largely responsible for the frequency and obstinacy of this trouble, but there seems to be such a diversity of opinion and so many different constitutional disorders are given as predisposing causes that we are forced to the conclusion that the subject is but little understood; for instance, some writers put it in the category of bone diseases, others ascribe its cause to wasting diseases, while rheumatism, gout, scrofula, malaria, tuberculosis, rachitis, and a host of others have each been brought forward as the cause. Some investigators claim to have proven it to be the result of a specific bacterium, and to have separated pure cultures, which in turn would produce a similar affection in animals, from which again pure cultures of the same bacteria were obtained; but biologists do not agree upon this point, notably, Miller, who has been unable to get the same result with his experiments and who questions the correctness of others' claims in this direction; therefore, in view of its doubtful origin, we can only be safe in recommending for general treatment any constitutional defect that may be present, as a precautionary measure, and then applying ourselves to the local sources of irritation.

Tartar about the necks of the teeth is the first step; then a careful following down of the denuded portions of the roots, and as thorough a cleaning as possible of the rough little deposits of sanguinary calculus nearly always present.

This is a matter of great difficulty, and its failure is probably the most common cause of recurrence of the disease.

Many instruments have been devised for the purpose, but none that I have used has given me such thorough satisfaction as small excavators ground flat. They can be prepared in a moment with a corundum disk in the engine, so that we have always at hand a scaler so small that it can be passed along the side of the root to the very end of the pocket with the least possible pain and laceration of the gum, besides being so sharp that the slightest sensation of roughness would be recognized by the fingers of the operator in a manner that would not be possible with a less delicate instrument.

In the application of local remedies three properties are necessary, antiseptic, acid and astringent.

Antiseptic because bacteria are always present, and to dissolve the particles of lime salts that may have and always do escape the mechanical cleansing; astringent, to reduce the inflammation and constrict the relaxed gum tissues. Hydrogen peroxid is a valuable wash for the purpose of syringing out the pockets; it destroys the pus, and at the same time acts upon the limy deposits in a most cleansing manner, by reason no doubt of the hydrochloric acid, which the preparations on the market have been found to contain. Dilute sulphuric acid, the aromatic preparation of sulphuric acid, the essential oils, and other remedies of a like nature are recommended. Pyrozone is also very highly spoken of.

Unfortunately, my supply of rabbits and guinea pigs has been somewhat limited, and I have therefore been obliged to confine my experiments to the pig in a higher state of development, according to the idea of the comparative anatomist.

During the last four years, since my residence in Duluth, a great deal of this trouble has come under my care for treatment, either because it is of a catarrhal nature, and the situation at the head of Lake Superior is particularly favorable to its development as to all catarrhal affections, or because having been constantly looking for it, more cases have come to my notice; but whatever the reason, it has appeared in every stage of development, apparently in much larger proportion than in my practice in other localities. The conclusions which I have to offer are therefore of an entirely practical nature.

First, in regard to its so-called incurability by reason of its relation to so-called constitutional causes.

My most obstinate cases have been almost invariably persons of robust stature, in whom apparently all of the predisposing constitutional affections were wanting, whose teeth gave little trouble from caries, every indication pointing to good structural development, and a healthful general condition.

A typical illustration of this class is a patient who came under my care in January, 1889, age 44 years; temperament a combination of bilious and sanguine; well-developed chest, good circulation, good digestion, regular habits, a vigorous, active business man; never had been seriously ill in his life, no catarrh of nose or throat, no rheumatic tendency, no skin disease, nor did a history of his case develop that any of his family had ever been, to his knowledge, affected by rheumatism, syphilis, scrofula, rachitis, or tuberculosis; and a most thorough examination of his urine failed to showed any normal condition pointing to Bright's disease or anything of that nature. Constitutional search abandoned, the examination of the arch gave but little better promise; the teeth were large with perfectly-developed crowns, each as nearly in its proper position as possible, the occlusion of the jaws correct, except where the affected teeth had elongated as a result of the disease; not a carious tooth among the number; all in place except the left superior third molar that had fallen a first victim to the disease; there was some accumulation of tartar, and its usual accompaniment of other deposits; the buccal roots of the right superior second molar were denuded their entire length. A loose flap of gum remained upon the buccal side of the first molar, but examination showed that the pus-filled pockets extended almost to the end of the root. The gums about the necks of the other teeth showed more or less pus upon pressure.

Treatment was continued at regular intervals for a period of two years, an antiseptic mouth wash prescribed and used regularly one or more times each day; the pulp canals of the two molars were cleaned and filled, for the continued formation of pus had finally reached the ends of the roots and destroyed the connection there; the pockets and necks of the teeth syringed with antiseptics, the loose gum removed by use of the lance and by absorption, until both molars had buccal roots denuded.

The first sign of improvement was a cessation of the discharge from about the necks of all the other teeth, and finally it was checked about the molars, and all around the edge of the gum at the line next to the affected molar roots there appeared what seemed to be a line of brighter colored new granulations.

I still see the patient every five or six months, and he continues the use of the wash, but apparently there is no return of the pyorrhea.

We have, all of us, had many such cases, and I have only gone into detail at the risk of wearying you, because it particularly illustrated some points I desire to call attention to.

We have seen that all ordinary methods of discovering constitutional predisposition failed, and the fact that the case yielded finally to local treatment would seem to indicate the cause as a local one. We know that the contagion theory is not borne out by the transmission by contact from one person to another, for if it were, undoubtedly husbands and wives would both be affected commonly instead of very seldom as reported, and yet, notwithstanding all this, I have under my care the two daughters of this patient, aged respectively twelve and fourteen years; they have each had quite severe inflammation of the gums, and quite recently I cleansed a pus-filled pocket at the neck and palatal root of a first molar in the mouth of the younger one.

Pyorrhea can undoubtedly be held in check and with continued proper care be prevented from reappearing, but whether or no it can be entirely cured, as is claimed by many, is a question not easily answered, because once severely attacked, even though the discharge be stopped, the parts never again regain that normal condition which should make them impervious to their destructive surroundings, and it is certainly a questionable difference whether a recurrence of the trouble be from the original infection or a fresh one; therefore, I hold that just so far as a condition of self-cleansing surfaces can be restored, in that proportion only can the cure be estimated; to this end, therefore, I recommend (notwithstanding instruction from high sources to save the gum margins) the removal of loose flaps of gum covering the pockets for I do not believe that they can ever be made to attach themselves to the separated surfaces again, and must of necessity afford a lodgment for infectional influences.

The loose teeth should be banded to firm ones, and the irritation of their movement in the sockets thus removed; also the occluding surfaces ground down until the excessive strain due to elongation be obviated.

The one final injunction before passing the subject, is to urge the importance of going to meet this trouble—not waiting until the pus-filled pockets are thrust upon us. Part of my regular examination is a pressure of my finger upon the gums inside and out all around the mouth, and it has been a matter of surprise to me how many of the apparently-unaffected cases showed that light-colored exudation, not pus but its almost certain forerunner.

This is the stage at which pyorrhea may certainly be cured, and to use a little Hibernian mode of expression, the best time to cure it is before it has begun.

In taking up the subject of infections from gangrenous tooth pulps and alveolar abscess, one is confronted by a most appalling array of dangerous possibilities; however, as the subject of this paper was suggested to my mind by a point raised in conversation with a prominent physician, I will quote his remark and then discuss from that standpoint. He said: "I believe in saving teeth and all that sort of thing, but it seems to me that dentists are too anxious to save teeth, and put on crowns to preserve roots that afterward cause serious trouble and are an injury to health instead of a benefit."

The undeniable force of this statement as generally applied struck me, and a perusal of medical literature showed, in almost every instance, wherever the general practitioner entered upon a discussion of diseases associated with the teeth the same idea governed his reference to the treatment of the mouth.

My answer to the above was a messenger next day, who brought the doctor in to see abscessed teeth treated, and some carious bone removed. I think he left convinced that correct surgical principles were applied in the mouth as elsewhere, and now, my answer to general writers is the bringing forward of well-known methods here urging their general distribution, that our best efforts may not be so often impeded by the suffering endured, and the serious complications that so frequently ensue before the dental practitioner is finally sought for relief.

Miller speaks quite bitterly of the custom of many physicians in disregarding dental diseases altogether as a factor in pathology, and says "it is as unjust to their patients as it is discreditable to their profession," but I do not agree with censure of the physician; the fault lies with dentists, and there is where the spur should be applied.

A gangrenous tooth pulp may be removed, the tooth canals disinfected by thorough drying with hot air or a heated wire and the use of antiseptics, preferably one that is not a coagulant (I use oil of cassia) and the danger of further infection removed, but when an apical abscess has formed, the destruction of the pus sac and the removal of the surrounding alveolar process as well become necessary.

An incision through the gum, followed by free use of a bur in the dental engine, quite readily effects the removal of the diseased tissues, then by enlarging the opening through the end of the root from the inside, medicaments injected into the opening in the crown can be freely forced through the end of the roots, and forced out through the gum, thus washing the entire affected surface perfectly clean.

A tooth thus treated is almost if not quite as fully under control as if it were extracted and in the hands of the operator, so far as cleaning it is concerned, and if the roots be immediately filled the wounded surface will heal up as readily as a simple cut.

Even the most chronic cases that have resisted other methods for years will yield to this treatment, provided, however, there be enough of the periosteum alive, but, of course, the destructive processes sometimes include also the entire surface of the periosteum, and the root being entirely dead, nature will not tolerate it, and her efforts to get rid of its annoyance will be continued.

A few fragments of necrosed bone are all that was left of that portion of the superior maxilla on the right side, extending from the central incisor to the tubercle, including the floor and part of the outer wall of the antrum, as well as part of the palatal process from the mouth of a young woman who suffered some three weeks before I saw her.

Removal of the necrosed bone, and the teeth in that region with it, the application of the engine and a large bur over all the roughened edges, washing with peroxid of hydrogen, oil of cassia and equal parts of peroxid and bichlorid of mercury 1-1000 gave almost immediate relief. A tonic was prescribed (for a slight formation of pus upon one of her thumbs gave reason to fear, as did also her color, that there was danger of pyemia), and also an antiseptic mouth wash, with instructions to use the latter freely. A rapid healing of the parts followed.

For three months a poor unfortunate was kept in one of our hospitals with a fracture of the lower maxilla that refused to unite. When he came to me there were three fistulous openings in his neck just below the angle of the jaw, into which the Sister of Charity packed cotton in masses as large around as my finger.

The fracture had occurred by reason of a blow from some heavy instrument—the man had been slugged and robbed—and search proved at the same time the pulp of a lower molar had been destroyed. Its removal, together with a portion of necrosed bone, syringing as in the last case, and in a few days the patient was discharged as cured.

Of very common occurrence is the infection of the maxillary sinus from apical abscess.

Many cases of so-called catarrh of the nose, throat and ear passages have had their orgin from this cause, and have been cured by treatment from the mouth in the following manner, viz: removal of the source of infection by extraction, or treatment of the tooth, and a free opening made through the floor of outer wall of the sinus, as low down as possible, to give better drainage and to allow therapeutic cleansing by forcing medicaments out through the communicating opening into the nose, and with the head thrown back let them run down the throat until they thus reach almost every portion of the exposed surface of the mucous membrane.

I believe it will soon be considered the correct method to open into and treat through the sinus every case of chronic catarrh of the nose.

The same lining membrane must and does transmit the infection to the antrum; the offensive secretions thus formed are held as in a pocket, where the usual treatment through the nasal passages can not reach them; what more natural, then, than the treatment described above?

No need to cite cases from practice of diseased antrum, we have all had too many of them, but I have given great relief in a number of instances where no purulent suppurative condition of the antrum was present, simply by the direct effect upon nasal catarrh that seemed to be otherwise incurable. One of my patients recently came to me in an extreme state of exhaustion, the constant presence in his throat and stomach of the discharges from his nose had prevented his eating or sleeping for some days and nights; relief was immediate, in fact, while he was still in the chair; and he made a business of eating and sleeping for some days afterward to make up (as he said) for lost time.

Peroxid of hydrogen I use until the passage into the nose is opened up, and after it a strong solution of salt and water is quite sufficient and very safe. Thorough drainage, however, is the most important step.

Syphilitic necrosis and lesions of the soft parts of the mouth require in addition to the surgical and antiseptic local treatment, the internal administration of iodid of potassium and mercuric bichlorid, for which, together with a proper general treatment, I always recommend them to the physician, knowing full well that we can each of us do better for the assistance of the other.

The presence of bacteria in such great variety and number in the mouth at all times must be looked upon as a menace, not only to the teeth in their relation to dental caries, but through their action as well upon the mucous membrane, in rendering it more susceptible to the germs of specific diseases, fevers, etc., upon the digestive tract, for many complaints of the stomach and intestines have been found to be caused by mouth bacteria and their waste products. Even the lungs are subject to this influence from the mouth, therefore its thorough disinfection becomes at once a matter of first importance.

Recognizing this fact, Miller, Black and others have prepared most carefully comparative statements of the strength of the various antiseptics used.

Miller particularly, has demonstrated that the rapid use of the ordinary antiseptic wash, unless accompanied by thorough cleansing is of comparatively little benefit, because at least several minutes are necessary to sterilize particles of food lodged between the teeth.

He recommends the following formula as one having the most rapid action:

℞ Acid thymic.......................... .25 grams.
Acid benzoic.......................... 3. grams.
Hydrargium bichlorid.................. .80 grams.
Tinct. eucalyptus..................... 15. grams.
Alcohol absolute......................100. grams.
Oilgaultheria.........................qt. xxv.
Mix.

Black's one, two, three mixture is:

℞ Acid carbolic.................................... i.
Oil cassia....................................... ii.
Oil wintergreen.................................. iii.

The wash that I have for some time past given to my patients is:

℞ Listerin.
Glycerin.
Acid carbolic.

Mix. Sig.: Dilute one-half teaspoonful in one-third glass of water, brush on the teeth and gums; hold in mouth and use on silk between teeth at night.

I give instructions to use full strength on the silk; in cleansing the mouth to hold it, and continue its use for several minutes consecutively.

This wash and the manner of using it has given my patients great satisfaction, not only as a means of checking tendency to caries, but also in fevers, such as typhoid, by relieving the unpleasant accumulations of mucus, and I believe has exerted an influence toward obviating the deteriorated condition of teeth, which almost invariably follows this class of diseases.

A physician who had noted its effect upon one of his patients got the prescription from me, and now uses it regularly in his practice.

The day is fast approaching when the treatment of the oral cavity will be a battle with therapeutic remedies rather than one of mere mechanical skill, and if I have seemed to give undue emphasis or wearied you with too careful note of little things, let me excuse myself with the honest statement that nothing in my professional experience has given me so much encouragement, so lifted me above the daily grind of bread-winning, or made me feel the great possibilities that are before us so keenly, as the bringing to light of some hidden source of trouble that perhaps for years had caused pain and distress, and being the instrument for its relief, and this can only be accomplished by looking for the little signs that have escaped notice by reason of their deceiving insignificance.

Surely 'tis an honest payment upon the indebtedness of our creation, and after all makes life seem worth the living.

Dr. Edgar Palmer spoke of a new tooth powder recommended to him by a physician for morbid conditions of the mouth, such as spongy conditions of the gums and fungous growths. The powder was quinine, and Dr. Palmer asked those present if they thought there was anything in it.

Dr. Talbot said there were three distinct conditions which were called pyorrhea alveolaris, and properly so-called, too, as each was shown by the flow of the pus from the alveolaris. The first was Riggs' disease, which results from deposition of tartar upon the teeth below the margin of the gums. The second is the condition described by Dr Ingersoll, characterized by the deposit of sanguinary or serumal tartar, and the third, of which variety I have cases at present, is a disease of the gums only, with no particle of deposit. I wish to bring out the necessity for systemic treatment for these cases. Unless the condition is good the disease will not be cured, and if the general system after the cure of the local disease is attacked by gouty, rheumatic or renal trouble the condition of the teeth will return.

We, as dentists, do not pay the attention to the mouths of our patients, which we should. It should be the rule to always examine thoroughly every mouth in which we have to do anything, and treat and clean it, putting it in a thoroughly healthy condition. This is a very important part of our work and we should get paid for it; otherwise we can not afford to do it. When a patient comes to me, where pockets are observed along the margin of the gums, I remove the tartar from the teeth and saturate the gums with clear tincture of iodine. After the second day, I

thoroughly overhaul the mouth and proceed with the treatment as recommended by the essayist. If, however, the teeth are loose, I can not treat successfully, except by removing the teeth. With the teeth extracted, it is a simple matter to heal up the gums and keep them in a healthy condition.

Dr. A. M. Benson asked how the iodine healed the gums?

Dr. Talbot said he did not know how, but knew that it did. He said that it was generally known that iodine would reduce tumors and swellings in all tissues, but we do not know how it acts.

Dr. Benson thought that if the tooth and gums were freed from all foreign substances the healing would come without medication.

Dr. Talbot said that in cases where there was no tartar on the teeth it was often possible to cure by systemic treatment, and spoke of cases in his own practice which he had successfully treated bv building up the system, omitting all local treatment.

Dr. W. A. Gudex said that Riggs' disease was perhaps the most puzzling and least promising of the troubles which dentists were called upon to treat. It might be described as an ulcerative pericementitis; sometimes the result of mechanical irritation, sometimes he thought the result of lead poisoning. He had noticed it in painters who had suffered from lead poisoning. He could not boast of much success in treating it. The only radical treatment was to remove the tooth or teeth affected.

Dr. J. Taft said that the treatment of diseased gums was very difficult, but he thought the treatment pointed out in the paper was suitable for a majority of cases. In many cases the general system is in such a state that it is impossible to cure the local disease without systemic treatment; the system must be toned up. In other cases the general condition is so hopelessly poor and defective that it can not be brought up to a state in which there would be hope of success. In such cases the only thing to do is to extract. Then there are cases where the general system is so good, is so well nourished, and has such recuperative power that if the irritant be removed the part will be restored. Remove all necrotic tissues either by an instrument, or by an escharotic, or by some other remedy such as iodine or pepsin; these seem to break down the necrotic substance and to stimulate the circulation which carries off the broken down, useless tissue. It is necessary to remove everything which will be an injury or an irritant. Simply pressing the finger on the part several times a day will be beneficial, as the pressure drives the blood away, and upon relieving the pressure there is an influx of new blood and an increased circulation which will help to carry off the diseased matter.

Robinson's remedy acts well as an escharotic, and if used as directed will prove efficacious in a large majority of cases. A dentist should always know just what effect he should expect from every remedy he uses, and why he uses the particular substance to effect the purpose.

Dr. Vida A. Latham spoke of the structure of the peridental membrane, and said that it had never been properly described. She denied that there was such a membrane, but said that it was the same as the periosteum, and that there was no reason just because it ran over the root of the tooth that its name should be changed. The descriptions in the text-books of this membrane vary; some say that it is an elastic membrane and others describe it as a ligament; she thought it showed careless writing in the books when they varied so. She thought a good name for pyorrhea alveolaris would be osteo-periostitis, meaning an inflammation of the bone and tissues surrounding the bone. These affections do not differ from similar inflammations in other parts of the body. Whenever found they are very hard to treat, as they usually occur in persons of poor organization, whose vital force is low and recuperative power almost nothing. In such cases the very structure of the bone is poor.

Dr. G. V. I. Brown said that his purpose in describing the case in his paper was to bring out discussion, as the case was one of perfect physical health, and he also wished to call the attention of the members of the Section to the fact that the daughter of the patient was troubled with the same disease, showing or appearing to show, that heredity was a factor in some cases.

